# Supplementary material for: A model for time interval learning in the Purkinje cell
Source: PLoS Comput Biol. 2020 Feb 10;16(2):e1007601. doi: 10.1371/journal.pcbi.1007601 (PMC7034954; doi:10.1371/journal.pcbi.1007601)
Supplement: S1 Table — (PDF) [file pcbi.1007601.s001.pdf]

Supporting information

S1 Table. Values for all parameters and coefficients of the model.

Calcium Store		
**Parameter**	**Value**	**Units**
α	1	ms^{-1}
β	1	ms^{-1}
γ	1	$\frac{\mu M}{ms}$
h	2	μM
n	4	-
k_1	2	ms^{-1}
k_2	0.01	ms^{-1}
B_{Ca}	0.01	$\frac{\mu M}{ms}$

Gi alpha subunit		
**Parameter**	**Value**	**Units**
a	0.0024	μM
b	0.0065	ms^{-1}

PKA Pathway		
**Parameter**	**Value**	**Units**
r_{1bl}	1e−7	ms^{-1}
r_{2bl}	0.0005	ms^{-1}
r_{ca}	0.2	ms^{-1}
r_{cag}	0.05	ms^{-1}
r_g	1e−6	ms^{-1}
k_7	0.002621	ms^{-1}
k_{a7}	1.439e−5	$(\mu M ms)^{-1}$
k_8	0.0501378	ms^{-1}
k_{10}	8.015779e−4	ms^{-1}
k_{13}	2.29e−13	$\frac{\mu M}{ms}$
k_{14}	4.7e−6	ms^{-1}
k_{15}	8.98e−11	ms^{-1}
K_{ca}	1e−5	μM
k_m	40.8587	μM
k_{Gi}	0.0002	mM

G protein-coupled receptor		
**Parameter**	**Value**	**Units**
K_g	0.01	μM

RGS Protein		
**Parameter**	**Value**	**Units**
r_{3bl}	0.1	ms^{-1}
r_4	0.0005	ms^{-1}
r_5	0.001	ms^{-1}
r_6	0.1	ms^{-1}
