# Supplementary material for: A model for time interval learning in the Purkinje cell
Source: PLoS Comput Biol. 2020 Feb 10;16(2):e1007601. doi: 10.1371/journal.pcbi.1007601 (PMC7034954; doi:10.1371/journal.pcbi.1007601)
Supplement: Attachment — Submitted filename: Response to reviewers_editor.pdf [file pcbi.1007601.s002.pdf]

Dear Editor,

Thank you for your positive feedback and decision to invite us to re-submit a revised version of our manuscript “A model for time interval learning in the Purkinje cell”.

We have revised the paper in accordance with the comments and the revisions made are detailed point-by-point below. We hope that the revisions will be considered appropriate and that the manuscript will now meet with your acceptance.

With best regards,
Daniel Majoral, Ajmal Zemmar, Raul Vicente; Tartu and Vancouver.

Reviewer #1

Comment 1: I thoroughly enjoyed reading this short but clearheaded manuscript.

The paper introduces a novel and insightful sub-cellular model that should account for paradoxical results from eye-blink conditioning experiments. The introduction is well argued and brings in relevant experiments. The suggestion that calcium oscillations in micro-domains is a component of learning is interesting and timely.

I did miss reference and discussion to recent molecular modelling results about the graded signal of the CS on Purkinje cells [1] which are aligned to the effects of calcium dynamics in the manuscript. Also, I would like to see a discussion of the role of signalling variability (CS duration / CS amplitude / firing frequency / dendritic diameters [as in 2]) on the molecular and circuit mechanisms.

[1] C. G. Zamora Chimal and E. de Schutter, “Ca2+ Requirements for Long-Term Depression Are Frequency Sensitive in Purkinje Cells.,” Front. Mol. Neurosci., vol. 11, p. 438, 2018.

[2] H. Anwar, C. J. Roome, H. Nedelescu, W. Chen, B. Kuhn, and E. de Schutter, “Dendritic diameters affect the spatial variability of intracellular calcium dynamics in computer models.,” Front. Cell. Neurosci., vol. 8, p. 168, 2014.

Response:

We thank the referee for the comments and the 2 references on the detailed modeling of calcium dynamics. How calcium signals exactly encode information and how this can be read is still an open question. Probably, it is the case that the information in calcium signals is encoded in a diverse set of features including the frequency and the total number of pulses but the details of how different molecules can read out this information is still uncertain.

In reference [1], the model of CaMKII response is sensitive to the frequency of the Ca2+ signal and LTD is induced by a specific number of Ca2+ pulses. Another interesting paper shows that the number of calcium spikes governs NFAT translocation [Wild, A.R., et al. Cell Reports, 2019]. We have added both references to the model limitations to remark that calcium spike counting seems more robust than simple amplitude summation as a read-out (see lines 304-312).

The role of signal variability on the molecular and circuit mechanisms is an important aspect. The complexity of the topic due to the large number of factors that can affect the signal and that would need to be explicitly modelled, it is however difficult to draw conclusive statements from these thoughts. We have included our thoughts in the discussion on this but find it difficult to discuss this on a more solid basis as the variables for the discussion are yet largely unknown.

Comment 2: In the discussion (line 211) the authors equate Purkinje cell interstimulus interval learning with learning of eye blink delay. I am unaware (but happy to learn) of studies that have tested minimum interval learning with electrical stimulus. I missed extensive references that would be required to back up with literature the problems 2 and 3.

Response:

Indeed the referenced paper (line 211) concerns eyeblink conditioning while the model concerns only the learning inside the Purkinje cell. There exists a paper that studies the minimum interval

learning with electrical stimulus [Wetmore D.Z. et al, J. Neuroscience, 2014], although there is no extensive literature on the subject. Now, we have this reference in new line 218.

For problems 2 and 3 we have also added the appropriate references. In [Johansson, F. et al, PNAS, 2014] and [Jirenhed DA and Heslow G, The Cerebellum, 2011], it was reported the observation of a double peak with cells trained with a new interstimulus interval, and the lack of an intermediate transition from the old interstimulus interval to the new one.

[1] Wetmore DZ, Jirenhed DA, Rasmussen A, Johansson F, Schnitzer MJ, Hesslow G. Bidirectional plasticity of Purkinje cells matches temporal features of learning. The Journal of neuroscience: the official journal of the Society for Neuroscience 2014;34(5):1731–7.

[2]. Johansson F, Jirenhed DA, Rasmussen A, Zucca R, Hesslow G. Memory trace and timing mechanism localized to cerebellar Purkinje cells. Proceedings of the National Academy of Sciences. 2014;111(41):14930–14934.

[3]. Jirenhed DA, Hesslow G. Learning Stimulus Intervals—Adaptive Timing of Conditioned Purkinje Cell Responses. The Cerebellum. 2011;10(3):523–535.

Comment 3: Also, in the discussion, the (compelling) suggestion that multiple domains learn different intervals, needs to be complemented by a couple of statements how different intervals would not be overwritten (if the PF bundles would be the same).

Response:

In principle, multiple times could be learned at different spatial locations (even if the PF bundles would be the same) if the learning mechanism exhibits some degree of stochasticity (i.e. only a few percentage of the synapses learn at a given time), and hence different synapses would be available for learning different time intervals. Also, it might be possible that feedback from inferior olive controls when synapse times are erased, thus the control of which times are overwritten would come from outside the Purkinje cell.

While our model is generative of the time-delayed response, other models relying on pre-existing mechanisms with the right temporal characteristics being selected (rather than generated) could also provide a way for the non-overwriting of different intervals.

Comment 4: Finally, concerning the limitations of the model (line 275):

- you are simulating simple and complex spikes but looking at simplified calcium and G protein mechanisms. The model limitations (line 275) should mention that the signals from inferior olive cells are not exactly synchronous nor particularly well timed.

What would happen to these results if say, 100 Purkinje cells were connected to 10 Inferior Olive cells with timings that are not precise nor reliable? If these olivary cells are synchronous and coupled, can you still conclude that the "yet to be understood mechanism" resides exclusively in the Purkinje cell?

Response:

These are relevant questions and we thank the referee for the suggestions. In the paper we did not mention the inferior olive despite its important role. Following the reviewer's thoughtful advice, we have added a comment on this aspect in the model limitations (see lines 313-320).

Our model (describing one micro-domain in a single Purkinje cell) is robust to small variability in the timing of the US. That is, the final learned time interval will converge near the average of the CS-US time intervals presented to the cell. At another level, inferior olive cells with timings that are not precise, nor reliable could certainly affect the pause response of the multiple Purkinje cells. However, if the imprecision of the IO cell's timing is only due to variability and not bias, then the overall output from a large set of Purkinje cells would reflect properly the time: When summing all the responses the pause maximum will correspond to the interstimulus interval time. Thus, ensembles of Purkinje cells can be robust to the variance of times (unless it is really high and the pause of different cells do not overlap in time). If the time signals has some large bias (the average time from Olive cells differs from the US time), and this is different for different stimuli, then it is difficult to envision an efficient mechanism that can learn the appropriate timing.

In respect to the second question, we believe that there exists a mechanism for associative timing inside the Purkinje cells, given the experimental evidence. If the inferior olive was perfectly synchronized or a perfect clock, still the Purkinje cell would have to integrate a large amount of sensory and motor information encoded by granule cells so it might be in a privileged position to make more complex temporal associations. However, even such mechanisms could be complementary to timing mechanisms occurring in the inferior olive. For example, to send proper teaching signals, the olivary nucleus should be able to discern time discrepancies between Purkinje cell output and the US. Our model is a simple proof of principle for intracellular models of timing mechanisms and does not exclude neither informs of other mechanisms that probably exist in the cerebellar system. We have now added this point to the Model limitations section in the Discussion.

Typos and grammar:

50: dot missing after 'consolidated'

61: 'in return' -> in turn

85: inverted quotes

133: "When through parallel fibers..." consider direct phrasing

Response:

We appreciate the input. We have corrected the grammar and typos mentioned by the referee.

Reviewer #2

This is an excellent paper! It is clear, succinct, well written. I like the fact that the authors themselves point to limitations and issues that should be explored further.

The paper is acceptable as it is. Nevertheless, I have some very trivial corrections and some suggestions for further thought. The latter should not be taken as requirements for rewriting but suggestions that the authors might find useful in improving the paper.

Minor corrections:

1. Page 1: The sense in which the Purkinje cell CR is timed should be explained. Readers not familiar with the area will not know if it is onset or offset or time to maximum amplitude that is timed.

2. Acronyms should be explained when they first appear (for instance IP3, PKA, PIP3).

3. L 50-51 “based in known” should be “based on known”.

4. L 52 delete “to”.

5. L 163. I do not understand this sentence. Should “with” be “to” instead?

6. Fig. 5 legend: number of previous pairings.

Response:

We thank the referee for the detailed input. We have corrected all points listed above and incorporated them into the manuscript. Regarding the first point, we have also added to the Introduction that the maximum amplitude and offset are timed (see lines 31-33).

Suggestions for improvement:

Comment 1: A crucial principle is the decoupling mechanism that will generate the same clock activity regardless of CS intensity or duration. A very brief CS of a couple of milliseconds has the same effect as a several hundred millisecond long CS (by the way, the correct reference is Jirenhed & Hesslow (2011) J Neuroscience 31:9070 –9074) but it is not clear how this is achieved. The text is to elliptic. I also found the illustration and description of the clock in relation to figure 2 confusing. The figure looks like an oscillating process with no trend, making it mysterious how a coincidence detection could detect how much time has passed. I assume that the clock involves a continuous change, such as increased calcium level, but this section could be expanded and explained more clearly. It might also be explained here why a long CS would not change the activity of the clock (decoupling). I realize that some of this is explained later in the paper but there is a point in introducing the basic principles in abstract terms and I think it can be done better.

Response:

We thank the referee to suggest these important changes to better clarify and illustrate the basics of the model.

Indeed, the decoupling mechanism should consider that the activation of subsequent calcium signal or its read out molecules become independent (to some extent) of the intensity and duration of CS. In principle, this could be achieved by different saturation effects. In our model this is achieved by the saturation of the effect of IP3 on calcium-induced calcium release. We now have added a brief explanation of how a saturation effect acts as a decoupling mechanism (see lines 97-99). We have also corrected the reference by Jirenhed & Hesslow.

We have changed Figure 2 and its caption to better convey a continuous change or trend, and thus reflecting better how the model works. Also, although the model does not perform calcium spikes counting, we have added to the discussion some remarks about calcium spike counting and how in principle it should be more robust than simple amplitude summation as a read out of the time interval (see lines 306-310).

Comment 2: There are a number of interesting features of conditioning that a good model should be able to explain. In particular, I think that the time course of the Purkinje cell CR, the pause response, should be dealt with. Just as with overt CRs, the onset latencies of Purkinje CRs do not vary much with varying CS-US intervals. What is timed is mainly the maximum amplitude and the offset. This is such a basic feature of the CR that it would seem to be a crucial test of the model that it can account for it.

Response:

As pointed out, we have focused on conveying that it is possible to learn time intervals with molecular mechanisms rather than to account for the precise features of the pause response. In experiments reported in [1], the authors considered interstimulus intervals of 150, 200, 300 ms and the estimated latencies to pause onset were of 13 ± 11, 48 ± 34, and 73 ± 18 ms, respectively. There is a lot of variance but the onset time reported are compatible with an increase as the interstimulus interval becomes longer. In our model the calcium signal also increases as the interstimulus interval becomes longer, therefore a simple readout of the calcium signal with a very low threshold that activates for example a GIRK channel should be able to explain the onset times. Later on the RGS molecule in the model can inhibit the same channel to start the ending of the pause, explaining maximum and offset times.

More generally, if the molecules involved in the onset latency differ from the ones involved in the offset or the maximum of the pause response, their different calcium integration rates and thresholds for activation could account for the time course of the pause response.

[1] Johansson, F., Jirenhed, D. A., Rasmussen, A., Zucca, R., & Hesslow, G. (2014). Memory trace and timing mechanism localized to cerebellar Purkinje cells. Proceedings of the National Academy of Sciences, 111(41), 14930-14934.

Comment 3: Another interesting fact is the change in CR latency that appears when the CS intensity is suddenly increased. Can they explain this intensity effect? (Svensson et al. 1997, Learning & Memory 3:105-115; Svensson et al. 2010, J Neurophysiol 103:1329-1336.)

Response:

We thank the referee for this interesting experimental observation. In our simplified model of a single microdomain, we have relied on a certain saturation in the IP3 released to explain the decoupling from electric currents for longer signals. Our model does not account for the spatial profile and extent of the activation of microdomains along the dendritic branch that could be an important factor to explain the effects on the CS intensity, especially if this is suddenly increased. How the spatial profile of the activation and its extent could interact with potential mechanisms of saturation (and hence of decoupling) is something we have not studied. Thus, beyond mere speculations, we may not be able to offer any theoretical account for the phenomenon at the moment.
